# Antibacterial Polysiloxane Polymers and Coatings for Cochlear Implants

**DOI:** 10.3390/molecules26164892

**Published:** 2021-08-12

**Authors:** Vlad Cozma, Irina Rosca, Luminita Radulescu, Cristian Martu, Valentin Nastasa, Cristian-Dragos Varganici, Elena-Laura Ursu, Florica Doroftei, Mariana Pinteala, Carmen Racles

**Affiliations:** 1 Department of Otorhinolaryngology, Faculty of Medicine, “Grigore T. Popa” University of Medicine and Pharmacy, 700115 Iasi, Romania; cozma.vld@gmail.com (V.C.); lmradulescu@yahoo.com (L.R.); critianmartu@gmail.com (C.M.); 2 Centre of Advanced Research in Bionanoconjugates and Biopolymers, “Petru Poni” Institute of Macromolecular Chemistry, 41A Grigore Ghica Voda Alley, 700487 Iasi, Romania; rosca.irina@icmpp.ro (I.R.); varganici.cristian@icmpp.ro (C.-D.V.); ursu.laura@icmpp.ro (E.-L.U.); florica.doroftei@icmpp.ro (F.D.); 3 Laboratory of Antimicrobial Chemotherapy, Faculty of Veterinary Medicine, “Ion Ionescu de la Brad” University of Life Sciences, 8 Sadoveanu Alley, 700489 Iasi, Romania; vnastasa67@gmail.com; 4 Department of Inorganic Polymers, “Petru Poni” Institute of Macromolecular Chemistry, 41A Grigore Ghica Voda Alley, 700487 Iasi, Romania

**Keywords:** *Streptococcus pneumoniae*, *N*-acetyl-l-cysteine, antibacterial, cochlear implants

## Abstract

Within this study, new materials were synthesized and characterized based on polysiloxane modified with different ratios of *N*-acetyl-l-cysteine (NAC) and crosslinked via UV-assisted thiol-ene addition, in order to obtain efficient membranes able to resist bacterial adherence and biofilm formation. These membranes were subjected to in vitro testing for microbial adherence against *S. pneumoniae* using standardized tests. WISTAR rats were implanted for 4 weeks with crosslinked siloxane samples without and with NAC. A set of physical characterization methods was employed to assess the chemical structure and morphological aspects of the new synthetized materials before and after contact with the microbiological medium.

## 1. Introduction

Cochlear implants are implantable medical devices that provide the chance to (re)habilitate hearing in deaf children and adults who have no benefit from conventional hearing aids. The importance of hearing in language development, verbal communication and social integration imposes cochlear implantation for those who suffer from bilateral severe profound congenital hearing loss, especially at younger and younger ages [1]. The current indications for cochlear implantation in adults are bilateral postlingual deafness, bilateral sensorineural hearing loss, bilateral profound hearing loss for high frequency with good hearing for low frequency, and, in some cases, asymmetric hearing loss with intensive tinnitus in the deaf ear. For children, the indication is for bilateral sensorineural hearing loss > 80 dB HL confirmed by hearing tests, after about 6 months of rehabilitation with the use of hearing aids [2]. According to the literature, in rare cases, however, there are complications related to the implantation and of these, infectious complications have an incidence ranging from 1.4% to 8.2% [3,4]. Even though the overall incidence is small, bear in mind that these infections occur mainly at a younger age and are, therefore, a problem that urgently needs to be solved. These are represented by infections at the incision site, necrosis of the musculocutaneous flap, and dehiscence of the surgical wound. The bacteria that cause cochlear implant related infections are most often represented by *Streptococcus pneumoniae*, *Staphylococcus aureus*, and *Pseudomonas aeruginosa* [5]. All of these elements provide potentially dramatic infections related to the implanted device. Despite therapeutic efforts to cure the infectious outbreak, explantation is almost always necessary [6].

Within this context, the most common strategies applied are the (1) antibiotic coating of implants and (2) covalent attachment of antimicrobial molecules on the surface of the implant to prevent bacterial adhesion, biofilm development, and avoidance of drug resistance to commonly used antibiotics [7].

The latest strategy used active molecules that are immobilized by covalent bonds on the implant surface, obtaining a bioactive implant that induced a decrease of the local toxicity and high resistance to bacterial colonization and biofilm formation [8]. This method is versatile and it can be applied for different implant materials, such as titanium alloys [9], glass [10], and silicon [11].

Such issues, presented above, also occur in the case of biointegrated electronics in vivo. Biointegrated electronics are widely applied for various in vivo applications, such as cochlear or cerebral implant [12], which should function in the humid, warm, and active environment of the human body and without developing infections on their surfaces. Thus, different types of materials have been proposed, such as a flexible semiconductor or silicon-based semiconductor materials. However, devices based on these rather rigid materials lead to an increased volume and adherence to the wavy shapes of various organs, which have limited their applicability. Those materials play an important role in preventing revision surgery or operations to replace the implant. In this context, their characteristics had to be defined. Thus, since 1992 [12,13], several requirements have been defined, such as the materials used must be biocompatible and must not introduce a risk of infection, and the insertion of the implant should be as noninvasive as possible, not cause additional damage, be efficient, and not damage the receiver/stimulator. In this context, up to the present, silicones are most commonly used for cochlear implants, recognized for their excellent biocompatibility, high flexibility, and high biostability. Nevertheless, evidence that the properties of silicones change once they are implanted in the body does exist [14,15]; in consequence, they are still under study to find a suitable design.

Different approaches have been proposed for a new generation of silicone materials that would give long-term antibacterial activity and prevent biofilm formation, preserving their biocompatibility. For example, recently, grafted hydrophilic polysiloxanes with *N*-acetyl-l-cysteine (NAC) with antibiofilm activity were signaled in the literature [16,17]. It is well known that *N*-acetyl-l-cysteine has weak antibacterial activity, but it is a strong biofilm inhibitor [17] by producing increased surface wettability and as a consequence, not allowing bacterial adhesion to substrates [18]. The most significant disadvantage of using NAC as a pristine molecule is its vulnerability to enzymatic degradation [19]. To overcome this problem, covalent attachment to a macromolecule or a material surface is a widely used but rarely investigated method.

In the present work, we present the synthesis of a series of new siloxane materials based on crosslinked polysiloxane bearing different contents of *N*-acetyl-l-cysteine and different degrees of crosslinking. It is important to note that the crosslinker is a siloxane copolymer, synthesized by us, containing thiol (SH) functional groups grafted on the main siloxane chain. The NAC grafting and the crosslinking reactions are based on thiol-ene photoaddition, following the method described previously [20]. The crosslinked siloxane materials were submitted to in vitro antibacterial tests against *S. pneumoniae,* and the results were compared with a silicone material without NAC, synthesized in the same manner as those with cysteine. In vivo tests were done on WISTAR rats implanted for 4 weeks with crosslinked siloxane samples without and with NAC. The prepolymers were characterized by ^1^H-NMR and the crosslinked polysiloxanes were characterized by different techniques, such as Fourier-transform infrared spectroscopy (FT-IR), differential scanning calorimetry (DSC), scanning electron microscopy (SEM), energy dispersive X-ray spectroscopy (EDX), wettability, and Raman techniques.

## 2. Results and Discussion

### 2.1. Synthesis of NAC-Modified Dimethyl-Methylvinylsiloxane Copolymers

**Scheme 1:**
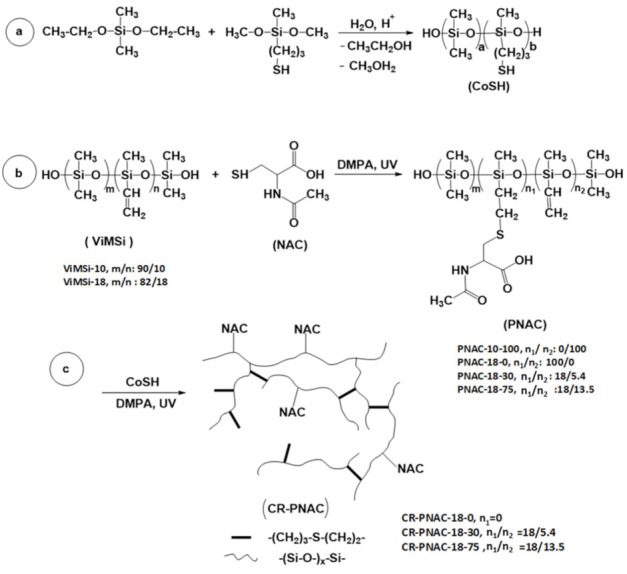
Chemical modification and crosslinking of NAC-modified polysiloxane: (**a**) preparation of the CoSH crosslinker; (**b**) preparation of PNAC copolymers by the modification of ViMSi copolymers with NAC, and (**c**) crosslinking of PNAC samples with CoSH.

The synthesis of NAC-modified dimethyl-methylvinylsiloxane copolymers with different percentages of vinylmethysiloxane units (PNAC-10-100, PNAC-18-30, and PNAC-18-75) was performed by thiol-ene photoaddition initiated by DMPA, following the method described previously [20,21], according to Scheme 1b.

As a model for structural characterization, all vinyl groups of ViMSi-10 with 10% of vinylmethysiloxane units (ViMSi-10, Scheme 1b) were reacted with NAC in the presence of DMPA under UV irradiation, giving completely modified PNAC-10-100 copolymer (Scheme 1b), while ViMSi with 18% of vinylmethysiloxane units (ViMSi-18, Scheme 1b) was partially modified with NAC, giving PNAC-18-30 and PNAC-18-75 samples (Scheme 1b). In order to obtain crosslinked films (Scheme 1c), suitable for biomedical purposes, PNAC-18-30 and PNAC-18-75 samples were UV crosslinked via the same thiol-ene addition reaction using an in-house prepared crosslinker, CoSH (Scheme 1a). CoSH synthesis was based on a co-hydrolysis reaction performed with a cation exchanger as a heterogeneous catalyst. The resulting compound had 6 mol% mercaptopropyl groups based on NMR and low molecular weight, as expected, i.e., Mn = 1400 g∙mol^−1^ (PDI = 1.5).

**Figure 1:**
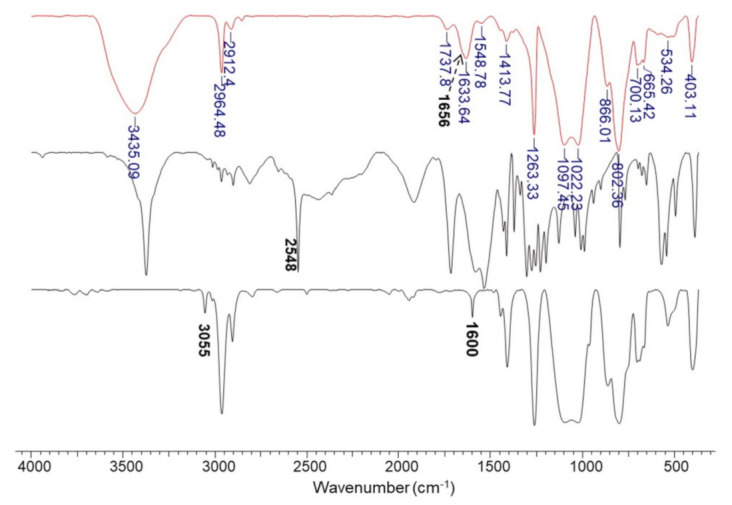
The FT-IR spectra of PNAC-10-100 (**red line**), NAC (**black line**), and ViMSi copolymer (**blue line**).

In Figure 1, the FT-IR spectrum of the PNAC-10-100 modified siloxane copolymer is compared with the spectra of the initial ViMSi copolymer and pristine NAC. It can be observed that the characteristic band for the SH bonds at 2548 cm^−1^ disappeared after the addition of SH function from NAC to the vinyl group of ViMSi copolymer, while the carbonyl bands suffered a rather important shifting, due to the modifications in H-bondings. The bands at 1736 and 1656 cm^−1^ are assigned to the C=O vibration mode of –COOH and –OC-NH, respectively [21]. In addition, the bands characteristic to the double bonds in the copolymer, at 3055 and 1600 cm^−1^, disappeared in PNAC-10-100, confirming the addition of NAC to the vinyl group. The polysiloxane signature (characteristic bands at 1263, 1097, 1022, and 802 cm^−1^) remained unchanged.

**Figure 2:**
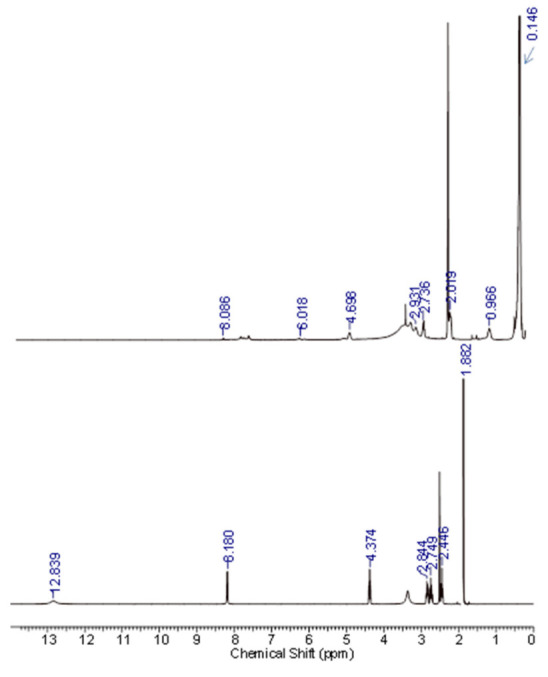
^1^H-NMR spectra of NAC (**bottom**) and PNAC-10-100 modified polysiloxane copolymer (**top**).

In the ^1^H-NMR spectrum of PNAC-10-100 (Figure 2), the SH chemical shift at 2.43 ppm in pristine NAC disappeared, as well as the majority of the vinyl signals of ViMSi at 5.5–6 ppm, showing the thiol-ene addition of NAC was practically complete. The other signals in the spectrum confirmed the formation of the new methylene groups (0.96 and 2.73 ppm) and preservation of the total substitution degree (i.e., ca. 10%) in the model copolymer.

### 2.2. In Vitro Tests

PNAC-10-100 copolymer and crosslinked siloxane samples (CR-PNAC-18-30, CR-PNAC-18-75) containing different percentages in NAC and CoSH crosslinker copolymer were submitted to in vitro tests against an *S. pneumoniae* strain specific infection to a cochlear implant [5], and afterwards compared with control samples (PNAC-10-100 and CR-PNA-18-0).

**Figure 3:**
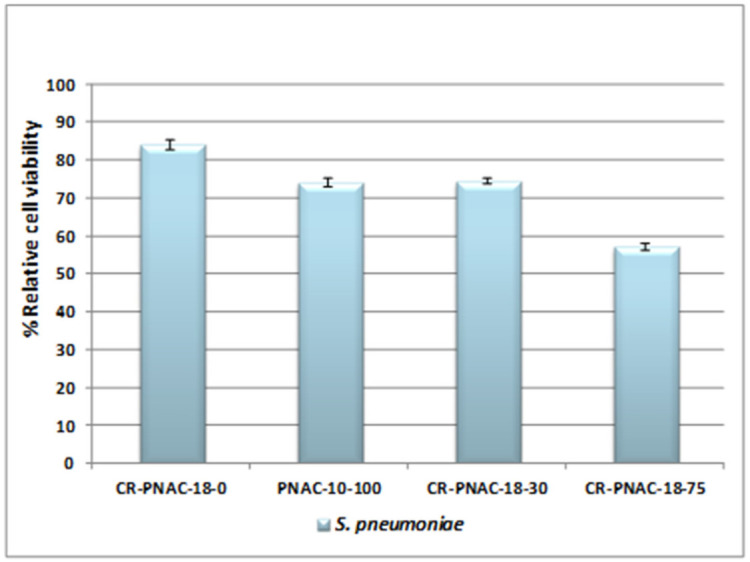
The antibacterial activity of the tested samples against *S. pneumoniae* CR-PNAC-18-0 control sample (without NAC and crosslinked with CoSH copolymer), PNAC-10-100, CR-PNAC-18-30, and CR-PNAC-18-75.

From the MTS assay (Figure 3) performed on the planktonic bacterial cells removed from the surfaces of the tested samples a decrease of the viability of the cells around 80% for CR-PNA-18-0 crosslinked siloxane without NAC, using CoSH crosslinker, was noticed, meaning that the nature of the crosslinking bridge plays an important role in the antibacterial activity against *S. pneumoniae* cells. It can also be observed that the viability of *S. pneumoniae* cells decreased in the series CR-PNAC-18-75 > PNAC-10-100 > CR-PNAC-18-30 > CR-PNA-18-0, indicating that even though the degree of crosslinking decreased with the increase of the content in NAC, the inhibition of *S. pneumoniae* cell proliferation is around 50% in the case of CR-PNAC-18-75 sample. It can also be concluded that with the increase of NAC percentage, the material became more efficient against *S. pneumoniae* cells. Taking into consideration these observations, the CR-PNAC-18-75 crosslinked polysiloxane sample was selected to be submitted to a more complex evaluation and to be compared with CR-PNA-18-0 as a control sample.

### 2.3. Compositional Stability of Samples before and after In Vitro Tests

**Figure 4:**
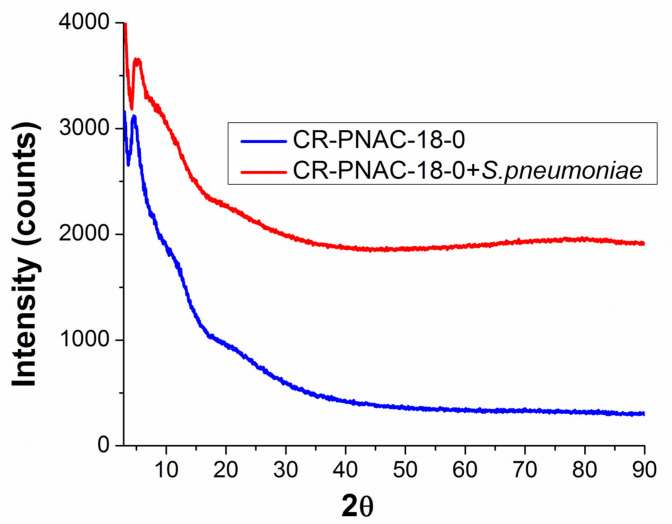
X-ray diffractogram (XRD) patterns of CR-PNAC-18-0 before (**red**) and after incubation with *S. pneumoniae* (**blue**).

**Figure 5:**
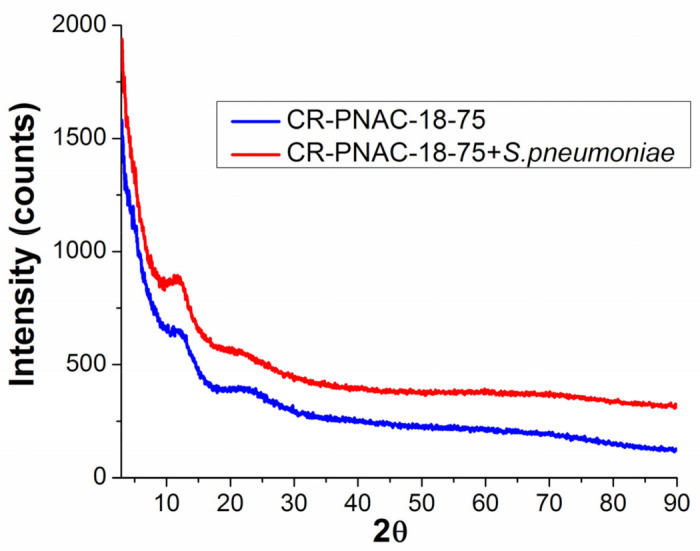
X-ray diffractogram (XRD) patterns of CR-PNAC-18-75 before (**red**) and after incubation with *S. pneumoniae* (**blue**).

In terms of XRD analysis (Figure 4 and Figure 5), all recorded samples (before and after bacterial incubation) were amorphous, with no visible differences between them. We can only discern the wide diffraction bands characteristic of polysiloxanes at approximately 14 and 20° 2θ, with some differences in intensity of characteristic bands after incubation with *S. pneumoniae* cells that may appear due to the biological attachment of cells after 4 weeks of incubation.

Moreover, the investigation of different modifications in the thermal transitions of the discussed materials (CR-PNAC-18-0 and CR-PNAC-18-75) was evaluated with the DSC method. The recording of the heat flow as a function of temperature induced composition-dependent transitions in, but not limited to polymer-based structures, playing a very important role in elucidating different material aging behaviors (microbiological, thermal, photochemical, etc.) [22,23,24,25].

**Figure 6:**
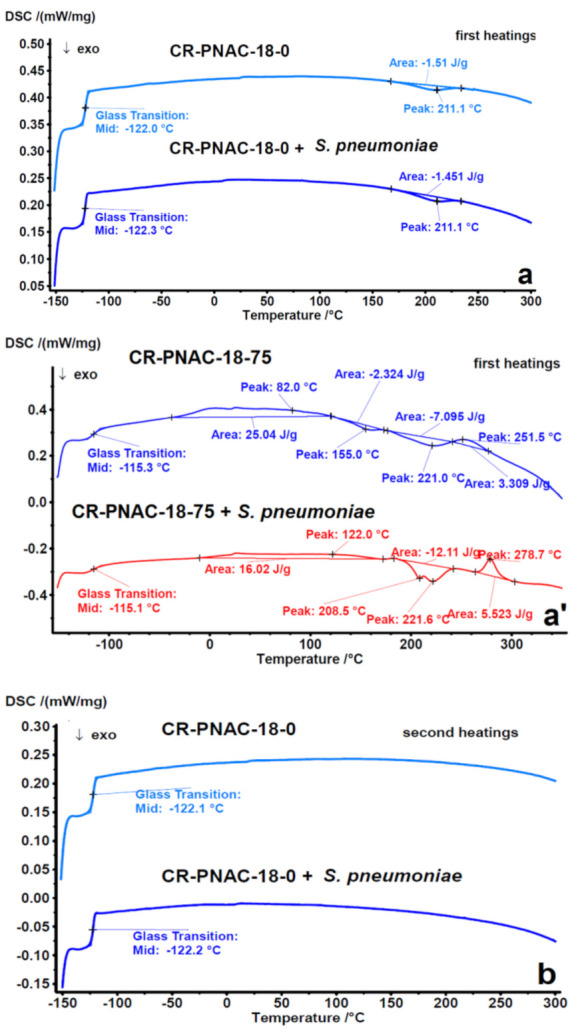

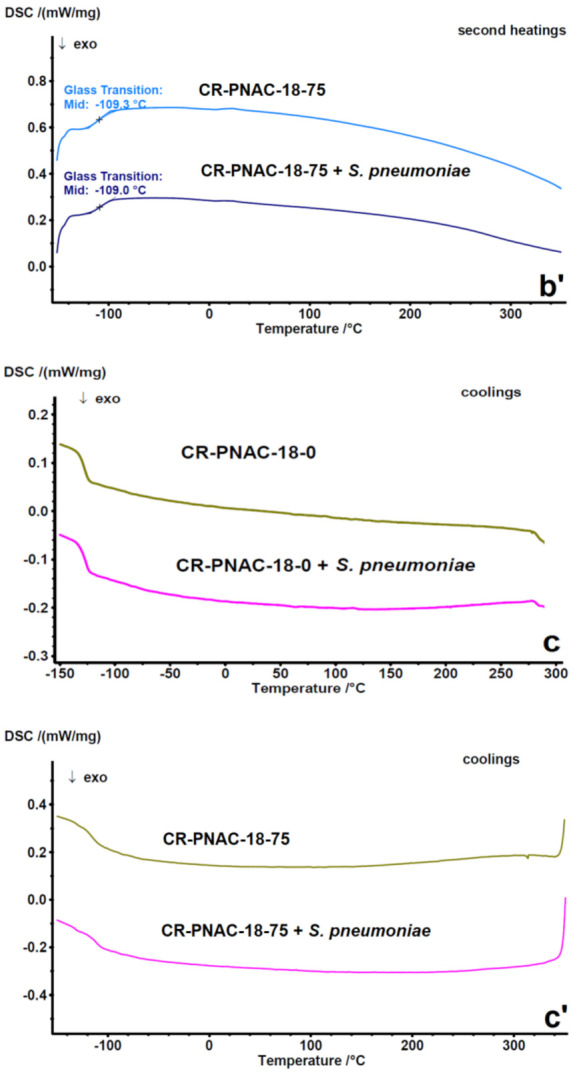
DSC curves of CR-PNAC-18-0 and CR-PNAC-18-75 before and after contact with *S. pneumoniae*: (**a**,**a’**) first heatings; (**b**,**b’**) second heatings; and (**c**,**c’**) coolings.

Figure 6(a–c,a’–c’) show the DSC heating-cooling-heating cycles CR-PNAC-18-0 and CR-PNAC-18-75 before and after contact with *S. pneumoniae*.

The broad endothermic peak present on the first heating curves of samples CR-PNAC-18-75 and CR-PNAC-18-75 treated with *S. pneumoniae*, ranging from around 0 °C to 200 °C, is due to moisture removal. From Figure 6, one may observe that the CR-PNAC-18-0 sample and CR-PNAC-18-0 after 4 weeks of incubation with *S. pneumoniae* exhibited a glass transition temperature (Tg) domain centered at −122 °C, typical for polysiloxanes. Thus, the T_g_s of these two samples reproduced almost perfectly, with no differences between them. Compared to the CR-PNAC-18-0 sample, the CR-PNAC-18-75 membrane before and after being incubated for 4 weeks with *S. pneumoniae* exhibited higher Tg values on the first heating curves, i.e., −115 °C. The fact that the Tg values registered for CR-PNAC-18-75 untreated and treated samples were identical on each heating curve confirms the homogeneity of the material. It should also be mentioned that single Tg values were registered, showing the absence of macroscopic phase separation, while the higher value as compared to pure PDMS-based materials is an additional confirmation of chemical modification with NAC. Both initial and treated CR-PNAC-18-0 samples exhibited an exothermic profile which reproduced at 211 °C and with similar enthalpy values (ΔH_cr_ = −1.51 J g^−1^ and −1.451 J g^−1^), most probably correlated with the crosslinking of traces of residual vinyl groups. The weak and almost perfectly reproducible exothermic profile seems to not have affected the Tg on the second heating of both systems (−122 °C). A different situation was noted for the initial and treated CR-PNAC-18-75, showing complex exothermic profiles at high temperatures (155–222 °C), which might be correlated with both thermal crosslinking of residual vinyl groups, and supplementary physical crosslinking by H-bonding favored by the increased chain mobility. For the initial CR-PNAC-18-75, two distinct exothermic profiles appeared at 155 °C and 221 °C with the corresponding ΔH_cr_ values of −2.324 J g^−1^ and −7.095 J g^−1^. For CR-PNAC-18-75 exposed to *S. pneumoniae*, the two exothermic peaks merged into a single profile with the first peak shifting significantly from 155 °C to 209 °C (ΔH_cr_ = −12.11 J g^−1^). The additional crosslinking would explain the higher Tg values registered on the second heating curves of the CR-PNAC-18-75 samples (−109 °C). On the first DSC heating curves of CR-PNAC-18-75 before and after exposure to *S. pneumoniae*, endothermal peaks were registered above 250 °C (252 °C with ΔH = 3.309 J g^−1^ for CR-PNAC-18-75 and 279 °C, ΔH = 5.523 J g^−1^ for CR-PNAC-18-75 + *S. pneumoniae*). However, these peaks were affected by thermal degradation. Moreover, no component of the materials has such a high melting temperature, and the presence of non-bonded NAC is excluded based on the XRD spectrum showing no crystalline compounds. In addition, no corresponding crystallization profile was observed on the CR-PNAC-18-75 cooling curve. Rather, a possible explanation could be the breaking of the H-bonds. Furthermore, there was a significant difference, in both temperature and enthalpy, between the endothermal degradation profiles of initial CR-PNAC-18-75 (252 °C, ΔH = 3.309 J g^−1^) and CR-PNAC-18-75 treated with *S. pneumoniae* (279 °C, ΔH = 5.523 J g^−1^). Such an important difference in thermal behavior between the sample before and after exposure to *S. pneumoniae* clearly indicates some kind of interaction of the copolymer with the culture medium triggered by the NAC organic groups. Therefore, it is most likely that the thermal degradation of the polysiloxane chains of CR-PNAC-18-75 was catalyzed by a component from the culture medium since different kinds of impurities are known to influence the polysiloxanes thermal degradation [26].

Overall, the analysis of the thermal behavior of the synthesized samples has no significance for the envisaged practical application, but it can show indirectly that the biological testing medium affected the behavior of the material during heating. The fact that enzymes degrade NAC is well established [17], while the enzymatic and pH-dependent degradation of PDMS in physiological conditions is considered insignificant [27,28]. The structural integrity of the material at ambient temperature after exposure to *S. pneumoniae* is proven by XRD analysis. Thus, we suppose that the “impurities” retained on the surface might generate, during heating, species which would have a catalytic role in enhancing the thermal degradation.

The different behavior of the modified films in presence of bacteria is surely influenced by the surface properties, i.e., contact angles and surface energy. Indeed, the dynamic water contact angle of the CR-PNAC-18-75 sample was measured by tensiometry (Wilhelmy method) and the found values were lower than those of pure PDMS, i.e., advancing and receding contact angles θ_a_ = 95.1° and θ_r_ = 57.8° (vs. 112° and 85°, respectively for PDMS). Both the relatively low θ_a_ for a silicone-based material and larger hysteresis (CAH = 37.3°) were normal, given the chemical modification with a hydrophilic group. The surface free energy (γ_S_) can be calculated from the contact angles [29] with Equation (1), where γ_L_ is the surface tension of water, i.e., 72.8 mN·m^−1^.

(1) $$\gamma_{s}^{\mathrm{tot}}=\frac{\gamma_{L}{\left(1+{\cos\theta }_{a}\right)}^{2}}{\left(2+{\cos\theta }_{a}+{\cos\theta }_{r}\right)}$$

The obtained value for CR-PNAC-18-75 was 24.7 (mJ·m^−2^), significantly increased compared with PDMS (16.5 mJ·m^−2^) [30]. The surface energy strongly affects bioadhesion. It seems that the adhesion minimum in water would be obtained at surface energy equal to the dispersive component of the surface energy of liquid water, i.e., 22 mN·m^−1^ [31]. This could explain the significantly lower attachment of the bacterial colony on CR-PNAC-18-75, proven by the SEM method, presented below.

**Figure 7:**
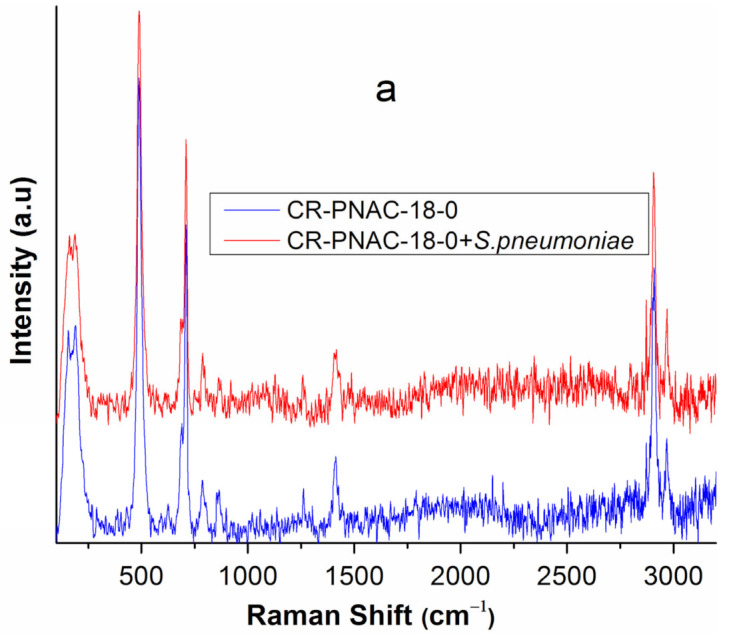

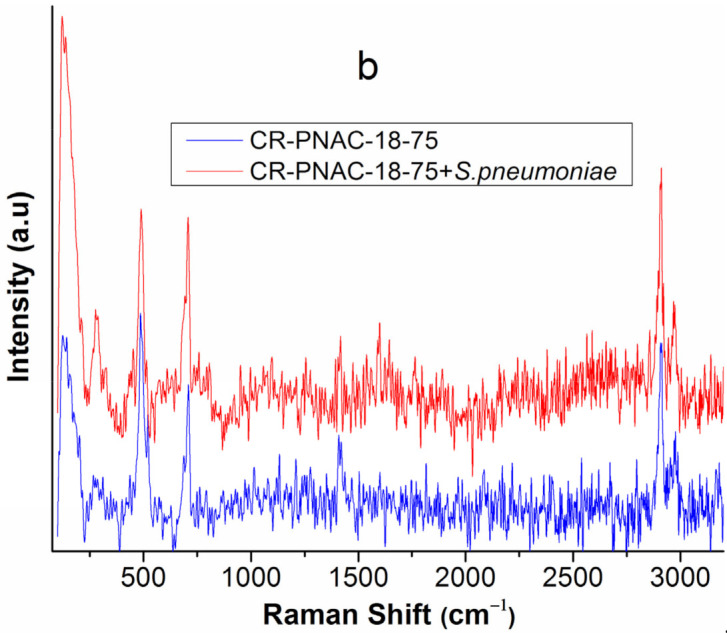
Raman spectra of (**a**) CR-PNAC-18-0 and (**b**) CR-PNAC-18-75 before and after bacterial exposure.

The Raman spectra were analyzed either to confirm the presence of biofilm by the “fingerprint method” [32] or to determine the potential structural modification induced by microbial exposure. Regarding the control sample, no characteristic Raman peaks for degradation and biofilm formation after exposure to bacteria were observed *(*Figure 7a). In contrast, the CR-PNAC-18-75 Raman spectrum presented some differences compared with the unexposed sample that suggests that *S. pneumoniae* bacteria may induce structural modifications, proven by the appearance of a new band at around 1600 cm^−1^ corresponding to amide I vibrational mode and of a more intense peak at 286.06 cm^−1^ accompanied by an increase in the intensity of the band at 708.19 cm^−1^ (Figure 7b). These modifications support the hypothesis of an interaction with the culture medium, which could have as consequence the enzymatic degradation of the NAC molecules on the surface during exposure to bacteria, as suggested by other authors [33].

### 2.4. In Vivo Tests

Following the results obtained from the in vitro test, it was observed that the best efficacy against *S.* *pneumoniae* was shown by the CR-PNAC-18-75 sample and therefore it was submitted for in vivo testing, after sterilization with ethylene oxide, by implantation for 4 weeks in young WISTAR female rats (mean age ≅ 4–5 weeks) weighing about 280–300 g each (see methodology in materials and methods, in vivo antimicrobial tests). Under the same conditions, the CR-PNAC-18-0 control sample was also tested in vivo.

### 2.5. Surface Characterizations of the Samples after In Vitro and In Vivo Tests

**Figure 8:**
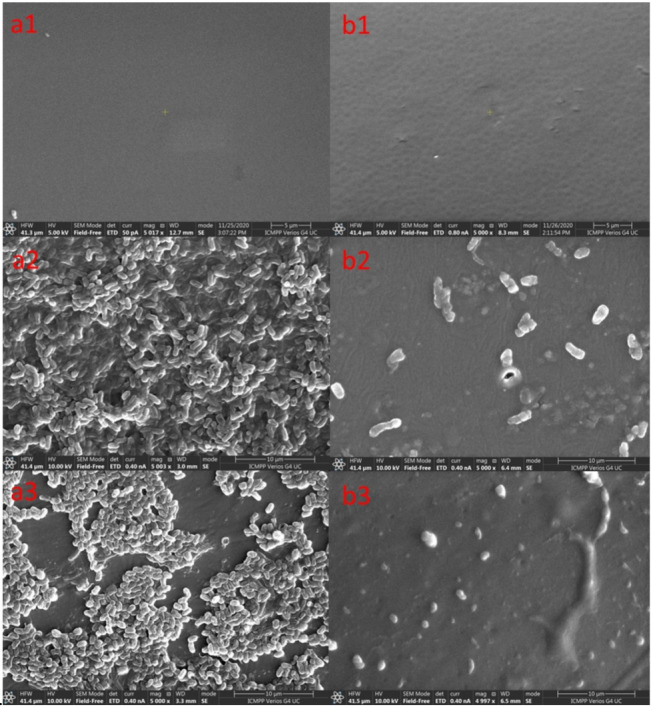
SEM surfaces images of CR-PNAC-18-0 (**a**) and CR-PNAC-18-75 (**b**) samples at 5 (**up**) and 10 (**below**) µm: (**a1**) and (**b1**), before incubation; (**a2**) and (**b2**) after in vitro incubation with S. pneumoniae for 4 weeks; (**a3**) and (**b3**) after in vivo incubation for 4 weeks.

SEM images obtained on the surfaces of the control sample (CR-PNAC-18-0) and CR-PNAC-18-75 sample before and after incubation with *S. pneumoniae* in vitro and in vivo after implantation for 4 weeks on WISTAR rats are presented in Figure 8. From these images, it can be noticed that the pristine samples presented homogenous surfaces before inoculation (Figure 8(a1,b1)), while surfaces exposed to *S. pneumoniae* cells in vitro and in vivo for 4 weeks presented different amounts of bacteria on the surface. In this context, CR-PNAC-18-0 samples (Figure 8(a2,b3)) presented pneumococcal biofilms with distinct aggregation and organization of the bacterial cells (diplococci and small chains) into clusters with empty spaces that will develop into pores. Moreover, the formation of extracellular matrix encasing the bacterial cells, a major component of the mature biofilm being remnants of dead bacteria, can be noticed [33].

Conversely, the images of the CR-PNAC-18-75 samples after 4 weeks of incubation with *S. pneumoniae* in vitro and in vivo (Figure 8(b2,b3), respectively) depict remnants from pneumococci lysing after being in contact with the membrane surface, the cell size appearing smaller than normal with no visible matrix. As an important conclusion, the CR-PNAC-18-75 sample after 4 weeks of incubation in vitro with *S. pneumoniae* cells and in vivo on WISTAR rats presented very good results, with only a few bacterial cells on their surfaces being found, without the tendency of biofilm formation. This is in agreement with previous reports showing that NAC-modified silicone materials were effective at eradicating mature bacterial biofilms [33].

Using the EDX analysis of CR-PNAC-18-0 and CR-PNAC-18-75 sample surfaces (Table 1), it was possible to evaluate their changes in the elemental chemical composition before and after in vitro and in vivo tests. It can be noticed that after in vitro tests, the surface of the control sample after 4 weeks of incubation with *S. pneumoniae* cells presented a significant increase in the percentage of nitrogen together with that of the oxygen and, also, the appearance of small quantities of other elements (such as sulfur, potassium, and chlorine), maybe as a result of the growth of the bacterial layer [34]. After in vivo tests, the surface composition of the control sample (CR-PNAC-18-0) was changed more evidently compared with its pristine one. The percentage of C and N increased, while the percentage of the other elements decreased (such as O, Si, S, and K). At the same time, the presence of P, S, Cl, and Na, in traces, was noticed. Moreover, the elemental analysis of CR-PNAC-18-75 surface was changed after in vitro and in vivo tests. The decrease in N and C percentages and the increase of O and S could be noticed, which were accompanied by the appearance of new elements, such as Na, Cl, and K. Probably, S does exist in less than 1% on the surface of the pristine CR-PNAC-18-75 sample and could not be detected by the EDX technique.

It is well known that, in silicone-base materials, the surface is generally enriched in Si. The very abrupt diminishment of the Si content of CR-PNAC-18-0 especially after in vivo test is in agreement with the SEM observation of biofilm covering most of the surface. In contrast, the NAC-modified polymer presented a less pronounced variation of Si content, again confirmed by SEM. Thus, the increase in S and O content in this sample after in vitro and in vivo experiments is mainly ascribed to changes in molecular conformation of the polymer at the surface during prolonged exposure to a wet environment.

## 3. Materials and Methods

### 3.1. Materials

2,2-dimethoxy-2-phenylacetophenone (DMPA, Merck, Darmstadt, Germany), *N*-acetyl-l-cysteine (NAC, Sigma-Aldrich, Saint Louis, Missouri, USA), diethoxydimethylsilane (Sigma-Aldrich, Darmstadt, Germany),dimethoxy-(3-mercaptopropyl)methylsilane (Sigma-Aldrich, Darmstadt, Germany), THF (Sigma-Aldrich, Darmstadt, Germany), and methanol (Sigma-Aldrich, Darmstadt, Germany) of high purity were used as received. Purolite CT-175 (sulfonated styrene-divinylbenzene cross-linked copolymer cationic exchange resins (St-DVB) with exchange capacity of 1.87 meq mL^−1^ was purchased from Viromet S.A. Dimethyl-methylvinylsiloxane copolymers (ViMSi) with 10 or 18 mol% methylvinylsiloxane units were prepared by a previously reported procedure [35,36,37].

### 3.2. Synthesis of CoSH Crosslinker Copolymer 

In a reaction vessel, 4.75 mL diethoxydimethylsilane, 0.25 mL dimethoxy-(3-mercaptopropyl)methylsilane, and 1 mL of distilled water were introduced and the mixture was purged with argon. Then, 0.1 g of St-DVB ion exchanger was added and the reaction was carried out at 50 °C for 4 h, then at room temperature for another 20 h. The cation exchanger was filtered out and the water and formed alcohols were removed by vacuum distillation. The resulted 1.5 g of CoSH copolymer (Scheme 1a) was kept under argon as a concentrated solution of 50% in THF.

For obtaining crosslinked films, partial modification of ViMSi with different amounts of NAC was carried out in the first step (Scheme 1b).

### 3.3. Synthesis of PNAC-X-Y

The PNAC-X-Y copolymers (P: polysiloxane copolymer; NAC: N-acetyl-L-cysteine; X: % vinylmethylsiloxane units in copolymer; Y: % NAC relative to X) were obtained similarly, as described below.

#### 3.3.1. Synthesis of PNAC-10-100

A measure of 0.5 g (6.65 mmol) ViMSi copolymer with 10 mol% vinylmethylsiloxane units (ViMSi-10, Scheme 1b) was dissolved in THF and to this solution, 0.11 g (0.67 mmol) of NAC dissolved in methanol was added, as well as 0.015 g DMPA. The mixture was UV irradiated at λ = 365 nm for 30 min. The solvents were removed under vacuum distillation and the product was thoroughly washed with water. The resulted PNAC-10-100 copolymer (Scheme 1b) was structurally characterized by ^1^H-NMR and FT-IR.

^1^H-NMR (CD_3_OD), δ (ppm): 0.15 (–SiCH_3_), 0.96 (-SiCH_2_-), 2.02 (-CH_3_), 2.73, 2.93 (-CH_2_SCH_2_-), 4.7 (-CH-).

#### 3.3.2. Synthesis of PNAC-18-75

A measure of 0.52 g (6.8 mmol) ViMSi copolymer with 18 mol% vinylmethylsiloxane units (ViMSi-18, Scheme 1b) was dissolved in THF and to this solution, 0.15 g (0.92 mmol) NAC (ensuring the modification of 75% of the vinyl groups) and 0.016 g DMPA dissolved in methanol were added. The mixture was UV irradiated (λ = 365 nm) for 60 min. The solvents were removed under vacuum distillation and the product (PNAC-18-75, Scheme 1b) was thoroughly washed with water.

#### 3.3.3. Synthesis of PNAC-18-30

To obtain PNAC-18-30 sample (Scheme 1b), the same procedure was applied as before, using the corresponding amounts of NAC and DMPA.

### 3.4. Preparation of Crosslinked Film

Preparation of crosslinked film: CR-PNAC-X-Y (CR: crosslinked; P: polisiloxane copolymer; NAC: *N*-acetyl-l-cysteine; X: % vinylmethylsiloxane units in copolymer; Y: % NAC relative to X). To obtain crosslinked films, the remaining vinyl groups of PNAC-18 with different amounts of NAC were crosslinked with CoSH.

Preparation of CR-PNAC-18-75 (Scheme 1c) is given as an example: The PNAC-18-75 copolymer obtained before was redissolved in a small amount of THF, filtered, and transferred into a Teflon Petri dish, then 0.2 mL of 50% THF solution of CoSH crosslinker and 4 mg DMPA were added. The resulting solution was UV irradiated for 30 min, then it was kept in a hood to slowly remove the remaining solvents. The resulting film (CR-PNAC-18-75, Scheme 1c) was peeled off after two days.

Preparation of CR-PNAC-18-30 and CR-PNAC-18-0 (as reference film without NAC) crosslinked polymers were obtained by the same procedure as described above, using the appropriate amounts of NAC and CoSH.

### 3.5. In Vitro Antimicrobial Tests

The antimicrobial efficacy of the materials was investigated by using in vitro adapted methods. A slightly modified Japanese industrial standard JIS Z2801:2000 which is based on a logarithmic number of live bacteria after 24 h of incubation was used, but in this case, the samples were incubated for 4 weeks, using a covering film to keep the thickness of the bacterial suspension. The antibacterial activity was determined against *Streptococcus pneumoniae* ATCC49619. The bacterial strain was refreshed in trypticase soy broth with defibrinated sheep blood for 24 h at 37 °C. The test surfaces were prepared as follows: each sample was placed in a Petri dish and sterilized at 254 nm for 15 min on each side.

The microbial suspensions were prepared with the reference strains in sterile solution to obtain turbidity optically comparable to that of 0.5 McFarland standards. Volumes of 0.2 mL from each inoculum were spread on the Petri dishes with trypticase soy broth with defibrinated sheep blood. The sterilized samples were placed on the plates, covered, and then incubated at 37 °C for 4 weeks in an atmosphere with 5% CO_2_. After incubation, the samples were rinsed repeatedly and pipetted to 1.5 mL Eppendorf tubes. In order to quantify the bacterial anti-adherence properties CellTiter 96sAQueous One Solution Cell Proliferation Assay, MTS (Promega) was performed, by following the protocol recommended by the manufacturer, using the solutions resulting from the previous experiment incubated in 96-well culture plates and incubated for 24 h at 37 °C. MTS Assay Kit uses a colorimetric method for the sensitive quantification of viable cells. The MTS reagent was then added to each well and, after 4 h incubation, the absorbance at 490 nm was recorded with an EnVisions plate reader (PerkinElmer). The experiment included four replicates and was repeated three times. All data were expressed as the mean ± standard deviation (SD). Statistical analysis was performed XLSTAT software [37].

### 3.6. In Vivo Antimicrobial Tests

The experiments were conducted on young WISTAR female rats. Seven young female rats (mean age ≅ 4–5 weeks) weighing about 280–300 g each were implanted. Out of those seven experimental animals, five of them were implanted with CR-PNAC-18-0 and two of them with CR-PNAC-18-75. Every CR-PNAC fragment was presterilized using the ethylene oxide sterilization method. The animals were anesthetized with ketamine (4 mg/100 g intramuscularly) and xylazine (1 mg/100 g intramuscularly). The rat was positioned in left lateral decubitus and the retroauricular region was shaved and cleaned with 10% povidone. The dissection for the auditory bullae was performed by a retroauricular approach. It is established that the posterior term describes the caudal direction and the anterior term describes the rostral direction. The incision comprised the skin layer and measured approximately 5 cm. The auricle was slightly pinched and oriented anteriorly. The first anatomical elements used as landmarks were the trapezius muscle, the great auricular nerve, and the parotid gland. These elements were easily seen through the transparency of the muscle fascia. It was necessary to palpate the right mandibular angle, thus easily identifying the parotid gland that hides the path of the facial nerve. The trapezius muscle was pushed posteriorly to expose the main trunk of the facial nerve. Specific care was required manipulating the trapezius muscle due to the presence at this point of a large blood vessel. The trajectory of the facial nerve was followed in the caudal direction until the periosteum of the auditory bullae was observed anteroinferior of the nerve.

Once the auditory bullae was accessible, the periosteum that covers the structure was stripped and then an opening was performed to gain access to the auditory bullae of the rat. To obtain access to the bullae, we used an otologic drill with a diamond head (1–3 mm in diameter). Subsequently, we prepared a socket for the test material that was comprised of musculocutaneous tissue placed on the back of the cervical area in connection with the created opening of the bullae. CR-PNAC-18-0 and CR-PNAC-18-75 were implanted in the pre-created musculocutaneous pocket, where the receptor-stimulator part of the device would normally be placed. After 4 weeks, all the samples were removed and submitted to morphological and physicochemical characterization.

### 3.7. Morphological Characterization

Surface morphology and elemental composition of the synthesized samples were evaluated with the help of a Verios G4 UC scanning electron microscope (Thermo Scientific, Brno-Černovice, Czech Republic) (SEM), equipped with an energy dispersive X-ray spectroscopy analyzer (Octane Elect Super SDD detector, Mahwah, NJ, USA) (EDX). After in vitro and in vivo incubation, the samples were inactivated at 121 °C for 15 min and then coated with 10 nm platinum using a Leica EM ACE200 Sputter coater to provide electrical conductivity and to prevent charge buildup during exposure to the electron beam. SEM investigations were performed in high vacuum mode using a secondary electron detector (Everhart-Thornley detector, ETD) at an accelerating voltage of 5 kV.

### 3.8. X-ray Diffraction (XRD)

X-ray diffraction analysis was performed on a Rigaku Miniflex 600 diffractometer using CuKα-emission in the angular range 3–90° (2θ) with a scanning step of 0.01° and a recording rate of 4°/min.

### 3.9. Differential Scanning Calorimetry (DSC)

The DSC analyses were conducted on a DSC 200 F3 Maia device (Netzsch, Selb, Germany). Around 5 mg of each sample were weighed and placed into aluminum crucibles. The crucibles were sealed shut with pierced lids. The measurements were undertaken in a nitrogen atmosphere (50 mL min^−1^ flow rate). The samples were heated and cooled at rates of 10 and −10 °C min^−1^.

### 3.10. Raman Spectroscopy

Raman spectra of samples, before and after microbial exposure, were recorded using an InVia Raman microscope (Renishaw, Wotton-under-Edgem, UK) in the 150–3200 cm^−1^ range. The Raman scattering was excited with a 785 nm near-infrared diode laser. Since the raw spectra acquired from samples presented a broad autofluorescence background, all the spectra were subjected to baseline correction using WiRE 3.2 software (Renishaw, Wotton-under-Edge, UK).

### 3.11. Surface Properties

Dynamic Contact Angle Measurements were done by the Wilhelmy method using a Sigma KSV700 Automatic Tensiometer (Helsinki, Finland).

### 3.12. Nuclear Magnetic Resonance (NMR) Spectroscopy

NMR measurements were done in CDCl_3_ at room temperature on a Bruker NEO-1 400 MHz spectrometer equipped with a 5 mm four nuclei (^1^H/^13^C//^29^Si) direct detection probe.

### 3.13. Fourier Transform Infrared (FT-IR) Spectroscopy

FT-IR spectra were registered on a Bruker Vertex 70 spectrometer (Ettlingen, Germany) in transmittance mode in the range 4000–400 cm^−1^ by accumulation of 32 scans, at a resolution of 2 cm^−1^.

### 3.14. Statistical Analysis 

Statistical analysis was done byusing XLSTAT | Statistical Software for Excel Available online [38].

## 4. Conclusions

Crosslinked new polysiloxanes based-materials with different contents in NAC and different degrees of crosslinking were prepared by a green process, meaning thiol-ene photoaddition initiated by DMPA. The obtained materials were submitted for in vitro tests against *S. pneumoniae* and in vivo tests on WISTAR rats for 4 weeks. It should be noted that as the structure of crosslinked polysiloxane contains a high NAC content, the material inhibits the formation of biofilms and decreases the number of bacteria adhering to its surface in both cases after in vitro exposure to *S. pneumoniae* or in vivo after implantation in rats for 4 weeks. Furthermore, it should be underlined that the structure of crosslinked polysiloxane without NAC is not affected after exposure to bacterial cells, while the materials with NAC in their composition are degraded maybe due to the enzymatic process of NAC. These features were demonstrated through XRD, DSC, and Raman methods.

After in vivo tests, when the CR-PNAC-18-75 sample was implanted on WISTAR rats for 4 weeks, the material surface showed bacteria in very small numbers and no biofilm formation was observed, making CR-PNAC-18-75 silicone material suitable for coating protection of biomedical devices, including cochlear devices.

## Figures and Tables

**Table 1:** SEM/EDX composition of CR-PNAC-18-0 and CR-PNAC-18-75 samples before and after in vitro and in vivo tests.

Element	CR-PNAC-18-0			CR-PNAC-18-75		
	Pristine	In Vitro	In Vivo	Pristine	In Vitro	In Vivo
**C**	29.06 ± 0.16	26.96 ± 0.26	35.36 ± 0.98	31.42 ± 0.01	30.36 ± 0.17	26.20 ± 0.91
**N**	0.67± 0.30	9.78 ± 0.03	2.82 ± 0.79	11.18 ± 0.15	10.76 ± 0.36	4.23 ± 0.43
**O**	27.72 ± 0.41	30.20 ± 0.88	26.11 ± 1.13	25.13 ± 0.47	28.35 ± 0.28	27.27 ± 0.43
**Na**	-	2.39 ± 0.07	0.395 ± 0.07	-	2.39 ± 0.88	1.13 ± 0.01
**Si**	47.60 ± 0.28	46.86 ± 0.79	33.93 ± 0.21	33.17± 0.12	27.10 ± 0.73	29.51 ± 0.12
**P**	-	-	0.12 ± 0.01	-	-	-
**S**	-	0.12 ± 0.25	0.07 ± 0.05	-	2.92 ± 0.14	3.31 ± 0.04
**Cl**	-	-	0.58 ± 0.70	-	0.83 ± 0.01	-
**K**	-	0.19 ± 0.02	0.09 ± 0.13	-	0.18 ± 0.01	0.19 ± 0.11

## Data Availability

Not applicable.

## Sample Availability

Samples of the compounds are not available from the authors.
